# Epidermal growth factor receptor double targeting by a tyrosine kinase inhibitor (Iressa) and a monoclonal antibody (Cetuximab). Impact on cell growth and molecular factors

**DOI:** 10.1038/sj.bjc.6602428

**Published:** 2005-03-08

**Authors:** J-L Fischel, P Formento, G Milano

**Affiliations:** 1 Oncopharmacology Unit Centre Antoine Lacassagne 33, Avenue de Valombrose 06189 Nice Cedex 2, France

**Keywords:** Iressa, cetuximab, epidermal growth factor receptor, drug combinations

## Abstract

Among the recent advances in the molecular targeted therapy of cancer, the applications focused on epidermal growth factor receptor (EGFR) are currently the most promising and the most advanced at clinical level. In view of the different modes of action of monoclonal antibodies and tyrosine kinase inhibitors (TKI), it is tempting to examine the effect of a combination between these two EGFR targeting approaches. It was the purpose of the present study to test this combination at experimental level by using two epidermoid human cell lines CAL 33 and CAL 39. As C225 (Cetuximab®) and ZD1839 (Iressa®) are, respectively, the most clinically advanced drugs in the category of anti-EGFR drugs, the experiments were performed using these two representative compounds. The combination of C225 and ZD1839 was antagonistic whatever the cell line considered. These antagonistic effects were corroborated by molecular changes in apoptosis (PARP) and EGFR signalling (phospho-p42–44). Drugs alone led to a diminution in EGFR levels, while their combination increased the cellular expression in EGFR. These data suggest that new and tempting treatment strategies on the EGFR target consisting in a double hit with a monoclonal antibody and a TKI must be considered with caution.

Epidermal growth factor receptor (EGFR) is a protein tyrosine kinase which plays a crucial role in signal transduction pathways that regulate key cellular functions such as survival and proliferation. Among the recent advances in the molecular targeted therapy of cancer, the applications focused on EGFR are currently the most promising and the most advanced at clinical level (Arteaga, 2001; Baselga, 2001; Ciardiello and Tortora, 2001; Yarden, 2001; Mendelsohn, 2002; Zwick *et al*, 2002). Considering the set of therapeutic tools targeting EGFR (Modi and Seidman, 2002), there are at present two well-identified emerging categories of drugs, the one characterised by monoclonal antibodies (Mabs) and the other by tyrosine kinase inhibitors (TKIs).

Mabs and TKIs clearly differ in their mode of action at target level. The primary action mechanism of C225, a chimeric Mab, is a competitive antagonism for EGFR. Independent of the phosphorylation status of the receptor, the EGFR–C225 complex is subsequently internalised (Fan *et al*, 1994; Prewett *et al*, 1996). The outcome of the EGFR–C225 complex following internalisation is not clearly documented, particularly regarding the stage between degradation and cell membrane recycling of the intact receptor. Tyrosine kinase inhibitors act on the cytosolic ATP-binding domain of EGFR by inhibiting EGFR autophosphorylation. Depending on the nature of the TKI, EGFR inhibition can be either reversible, as with ZD839 or OSI-774, or irreversible, as for instance with PD183805 (Noonberg and Benz, 2000; Ciardiello and Tortora, 2001; Denny, 2002). The irreversibility of the inhibition is due to covalent fixation of the drug at the ATP-binding site. In contrast to the approach using Mabs, the use of TKIs is not strictly specific for the ATP pocket of the EGFR; this can be explained by the fact that TKIs are all ATP competitors at the ATP site of the tyrosine kinases (Denny, 2002). Thus, for TKIs, some variable crossreactivity may exist between EGFR and other HER-B family members such as HER-2 (Arteaga, 2001).

In view of the different modes of action of Mabs and TKIs on EGFR, it is tempting to examine the effect of a combination between these two EGFR-targeting approaches. In addition, clinical studies indicate that the pharmacodynamics of Mabs and TKIs are not strictly superimposable with side effects as regards Mabs being mainly centred on the skin and, regarding TKIs, mostly related to the skin and to gastrointestinal intolerance (Thomas and Grandis, 2004). It was the purpose of the present study to test this combination at the experimental level by using two epidermoid human cell lines CAL 33 and CAL 39. As C225 (Cetuximab®; Herbst and Hong, 2002; Saltz *et al*, 2004) and ZD1839 (Iressa®; Ranson *et al*, 2002; Fukuoka *et al*, 2003) are, respectively, the most clinically advanced drugs in the category of Mabs and TKIs, the experiments were performed using these two representative compounds. Along with an analysis of the drug impact on cell proliferation, the present study included an examination of drug effects on apoptotic pathway (phospho-AKT and PARP cleavage), on MAP kinase pathway (phospho-MAPK) and on EGFR expression.

## MATERIALS AND METHODS

### Chemicals

All chemicals were obtained from Sigma Chemicals (Saint Quentin Fallavier, France) and were of the highest purity grade. DMEM and glutamine were obtained from Biowhittaker (Verviers, Belgium) and foetal bovine serum (FBS) from Dutscher (Brumath, France). Drugs were kindly provided: Cetuximab® (C225) by Merck (Darmstadt, Germany) and Iressa® (ZD1839) by Astra Zeneca (Rueil Malmaison, France), respectively.

For ZD1839, a 50 mM working solution in dimethylsulphoxide (DMSO) was prepared before use. Radiochemicals, ^3^H-thymidine and ^125^I-EGF, were provided by Amersham Biosciences (Orsay, France).

### Cell lines

One human head and neck cancer cell line CAL33 and one human vulvar cancer cell line CAL39 were used in the present study. Epidermal growth factor receptor levels (fmol mg^−1^ prot^−1^) were 33 800 and 6500 for CAL33 and CAL39, respectively (ligand-binding assay as previously published by us; Olivier *et al*, 1990). Both cell lines were maintained in DMEM supplemented with 10% FBS, 2 mM glutamine, 50 000 U l^−1^ penicillin and 80 *μ*M streptomycin in a fully humidified incubator (Sanyo, Japan) at 37°C in an atmosphere containing 8% CO_2_.

### Drug administration schedule

Drug effects were assessed using ^3^H-thymidine incorporation. We checked whether this assay compared well with the viability results obtained with the classical MTT test: the results were almost superimposable for ZD1839; it was not the case for C225, which exhibited acceptable dose–effect curves with the thymidine incorporation only. Experiments were performed in DMEM supplemented with 0.5% FBS. This low serum concentration was selected so as to reduce the presence of exogenous growth factors while ensuring a correct survival of cells without drug. Cells were seeded on day 0 in a 24-well microplate, 18 000 cells per well for CAL33 and 24 000 cells per well for CAL39. The cell seeding concentrations were chosen to allow exponential growth throughout the experiment. Drugs were added 48 h after cell seeding and applied during 96 h either alone or in combination. The range of drug concentrations was chosen according to the results of preliminary experiments in order to obtain the most complete single-agent dose–effect curves. Concentrations thus varied between 10^−11^ and 10^−7^ M for C225 and between 10^−8^ and 10^−4^ M for ZD1839. The assay method was essentially that described by Volm *et al*. (1970): after the end of the drug incubation time, that is, 120 h after cell seeding, [^3^H]dTHd was added (52 CimM^−1^; final concentration, 3 *μ*Ci ml^−1^) and incubation was continued for another 2 h. Plates were then cooled on ice. The experiments were ended by adding ice-cold 5% trichloracetic acid (TCA) and the wells were washed twice with TCA, then precipitated DNA was dissolved in NaOH 10% solution and counted on a liquid scintillation counter (Wallac, 1409 DSA). Results were expressed as the percentage of radioactive incorporation relative to a control without the drugs. Experiments were performed at distance in triplicate.

### Drug interaction analysis

#### Combination Index (CI) calculations

The cytotoxic effects obtained with the ZD1839/C225 combinations were analysed according to the Chou and Talalay method (1984) on Calcusyn software (Biosoft, Cambridge, UK). Interaction between ZD1839 and C225 was assessed by means of an automatically computed CI.

The use of the Chou and Talalay ‘CI’ requires a dose–effect curve, which reaches the zero value for survival. This was not strictly the case for the C225 dose–effect curve. For this reason, in a complementary analysis, we replaced the CI by the following ratio *R*: 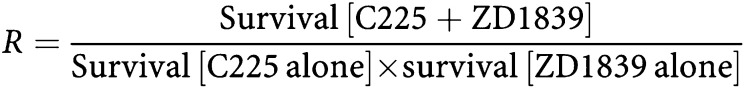 for example, if C225 at a concentration [c] and ZD1839 at a concentration [z] which, when given alone, have a 50% growth-inhibitory effect and, when given together (at these same concentrations, respectively, [c] and [z]), have a 75% effect, then in this case *R* will be: (1–0.75)/0.5 × 0.5=1.

Then, if *R*<0.8, the association is considered to be synergistic 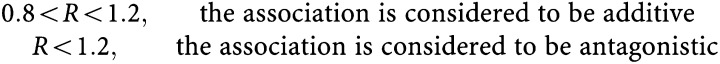


### Molecular factors

All experiments concerning the following investigated molecular factors were performed on CAL33 cells.

### Epidermal growth factor receptor measurement by ligand-binding assay

Epidermal growth factor receptor expression was assayed as previously described by us (Olivier *et al*, 1990). Cells were grown to 80–90% confluence, in 24-well plates, in 10% FBS–DMEM at 37°C. Then drugs (ZD1839 2 × 10^−6^ M, C225 10^−9^ M final concentration) were added and incubation was performed during 2 days. Cells were rinsed three times with 500 *μ*l RPMI 1640 containing 0.1% BSA at 2–4°C and incubated for 30 min with the same medium (500 *μ*l per well) at 4°C. Epidermal growth factor receptor content was determined by incubation in RPMI medium for 3 h at 4°C in the presence of increasing concentrations of ^125^I-EGF (0.01, 0.02, 0.04, 0.08, 0.12, 0.18, 0.2 nM) and with 0.2 nM of ^125^I-EGF with increasing concentrations of unlabelled EGF (0.05, 0.1, 0.2, 0.4, 0.8, 1.6, 3.2, 6.4, 20, 200 nM). Plates were put on ice to stop the reaction, the supernatant was removed, and cells were washed twice with PBS containing 0.1% BSA (4°C, 500 *μ*l per well). Cells were solubilised in 1 M NaOH at 37°C (500 *μ*l per well for 30 min). Radioactivity was determined by gamma counting. The number of receptor sites per cell (*N*) and the dissociation constant (Kd) were determined by Scatchard analysis (each point on the Scatchard plots was plotted in quadruplicate). Cell number was determined in wells run in parallel, by resuspending cells in 200 *μ*l PBS at room temperature and counting with a haemocytometer. Experiments were performed at distance in duplicate.

#### Western blot analyses

Cells were seeded in a 75 cm^2^ cell culture flask for 48 h in DMEM+10% FBS medium. They were then washed three times with PBS and incubated for another 48 h in DMEM medium without FBS in the presence of 2 × 10^−6^ M ZD1839 and/or 1 × 10^−9^ M C225 (concentrations at which drug maximum effect on thymidine incorporation was obtained). Then, at the end of drug exposure, serum (FBS, 10% final concentration) was added and cell pellets were collected at t0 (before addition of serum) and 5 and 30 min after addition of FBS. All cell pellets were stored at −80°C before analysis. Cell pellets collected at t0 were used for the measurement of cleaved PARP and EGFR; those obtained at 5 and 30 min were intended for the analysis of PAKT and Pp42–44. The expression of EGFR, HER-2, cleaved PARP, PAKT, Pp42–44 was measured using the Western blot technique. Briefly, equivalent quantities of protein for each drug combination were separated on a 12% SDS–polyacrylamide gel (EGFR and HER-2 excepted, for which a 7.5% polyacrylamide gel was used). After overnight transfer to a membrane, the proteins were revealed: for PAKT, the first antibody was rabbit polyclonal (Ozyme) 1/1000 in TBS 5% BSA using Jurkat cells as a positive control. For Pp42–44, the first antibody was of mouse origin kindly provided by CNRS Unit 6543 (Centre Antoine Lacassagne, Nice) 1/5000 in TBS 5% milk using A431 as a positive control. For PARP, the first antibody was of Rabbit origin (Ozyme) 1/1000 in TBS 5% milk. For EGFR, the first antibody was the mouse monoclonal Ab12 (Neomarkers) 1/1000 in TBS 5% milk using A431 as a positive control. After blotting with an adapted second antibody, proteins were revealed in a darkroom using ECL. All experiments were performed in triplicate.

## RESULTS

**Figure 1:**
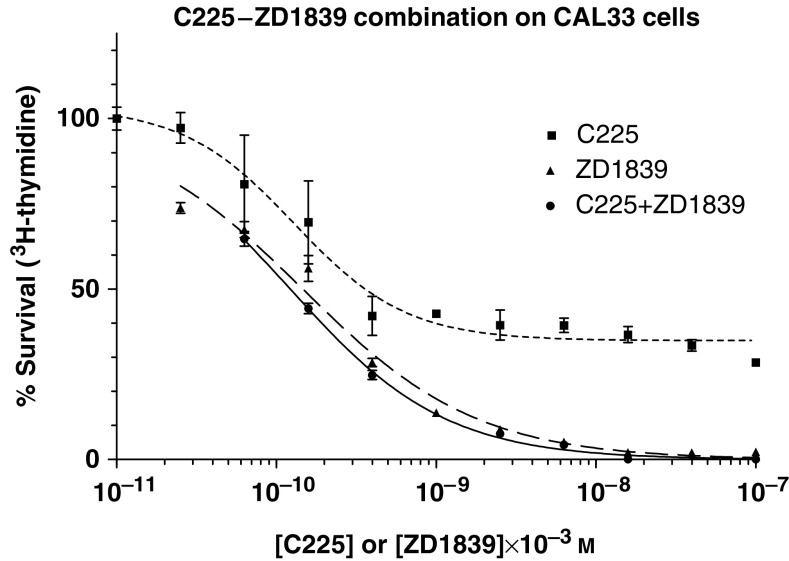
Dose–effect curves of C225 alone, ZD1839 alone and their combination on CAL33 cell line. Bars depict standard deviations from triplicate experiments.

**Figure 2:**
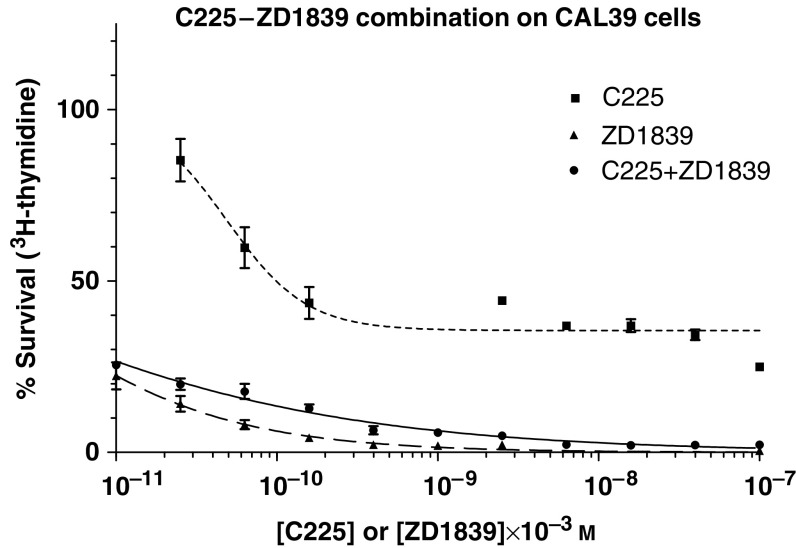
Dose–effect curves of C225 alone, ZD1839 alone and their combination on CAL39 cell line. Bars depict standard deviations from triplicate experiments.

The effects of C225, ZD1839 and their combinations are described in Figures 1 and 2 for CAL33 and CAL39 cells, respectively. Of note, and for both cell lines, C225 alone never led to 0% survival. The dose–effect curves of the C225+ZD1839 combination were either just under (CAL 33 cells) or even clearly above (CAL39 cells) the ZD1839 dose–effect curve. This direct observation suggests an antagonistic interaction between the two drugs. The examination of both *R* values and CIs confirmed that the combination of C225 and ZD1839 was antagonistic whatever the cell line considered (Table 1 ).

**Figure 3:**
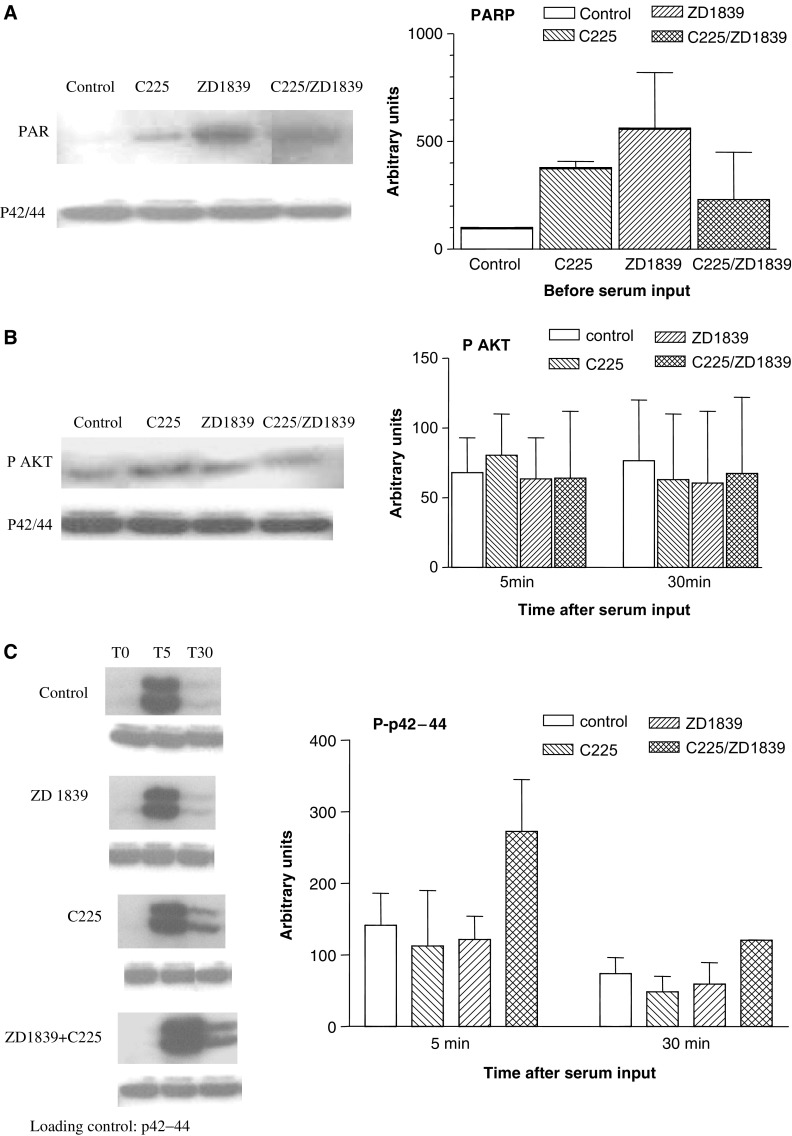
(**A**) Effects of C225, ZD1839 and their combination on PARP cleavage after the end of drug exposure and before serum input on CAL33 cell line. Bars depict standard deviations from triplicate experiments. (**B**) Effects of C225, ZD1839 and their combination on PAKT expression induced by serum input on CAL33 cell line. Bars depict standard deviations from triplicate experiments. (**C**) Effects of C225 and ZD1839 on the P-p42–44 expression induced by serum input on CAL33 cell line. Bars depict standard deviations from triplicate experiments.

The effects of C225, ZD1839 and their combination on PARP, PAKT and Pp42–44 are depicted in Figure 3. The impact of ZD1839 on PARP cleavage was more marked than that generated by C225 alone (Figure 3A). Of note, when the two drugs were combined, there was a smaller change in PARP as compared to the change with ZD1839 alone. This finding is in agreement with the data on cell survival indicating infra-additive cytotoxic effects resulting from the association of the two anti-EGFR drugs. There were no clear-cut effects of ZD1839, C225 or their combination on PAKT (Figure 3B). ZD1839 or C225 given alone downregulate Pp42–44 up to 30′ after serum addition; in contrast, combining them clearly enhanced this cell division-related pathway (Figure 3C). This observation corroborates the results on cell proliferation and strengthens the antagonistic interaction for cell survival between ZD1839 and C225.

**Figure 4:**
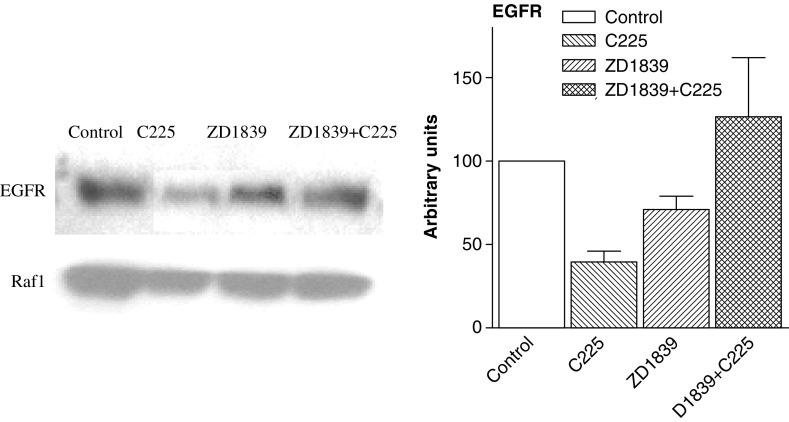
Effects of C225, ZD1839 and their combination on EGFR expression on CAL33 cell line. Bars depict standard deviations from triplicate experiments.

The apparent antagonism between the two drugs could be accounted for by a modification of EGFR sites. Epidermal growth factor receptor cell quantification and EGFR ligand affinity are depicted in Table 2 and Figure 4 for the different tested conditions. Drugs alone led to a diminution in EGFR levels as compared to controls, with a stronger effect for C225 as compared to ZD1839. In contrast, the combination of the two drugs increased the cellular expression in EGFR. This increase was observed with both EGFR protein measurement (Figure 4) and with the specific quantification of EGFR receptors (Table 2).

## DISCUSSION

In the current search for new therapeutic targets in cancer, growth factors and their receptors represent a field of active investigation both at preclinical and clinical levels. The EGFR receptor is significantly overexpressed in solid tumors and constitutes an important target for the development of the new targeted anticancer drugs (Baselga, 2001; Mendelsohn, 2002). There are two different categories of compounds in the current arena of active drugs targeting EGFR with monoclonal antibodies on the one hand and TKIs on the other. C225 (cetuximab) is the most clinically advanced representative drug in the family of monoclonal antibodies targeting EGFR (Herbst and Hong, 2002; Saltz *et al*, 2004). ZD1839 (Iressa) is one of the EGFR-specific TKIs currently under active clinical investigation (Ranson *et al*, 2002; Fukuoka *et al*, 2003). Although C225 and ZD1839 target the same EGFR receptor, there are some differences between these two drugs. First, they interact with different domains of the EGFR protein with C225 recognising the extracellular part of EGFR and ZD1839 acting on the intracellular ATP pocket of the tyr kinase entity. As the biological activity of ZD1839 may be attributed to its antityr kinase action, the exact mechanism of action of C225 is perhaps more complex. Its activity is partly related to its interaction with the EGFR and partly related to antibody-directed cell cytotoxicity as described for Herceptin (Clynes *et al*, 2000). C225 is administered by i.v. injections, while ZD1839 is an oral drug. The pharmacodynamics of the two drugs, although superimposable regarding adverse cutaneous effects, differ as regards digestive toxicity, which is mainly observed during ZD1839 treatment (Thomas and Grandis, 2004). Of interest, the two drugs may possess different resistance mechanisms, since it has recently been reported that the clinical efficacy of Iressa in lung cancer patients was related to the presence of activating mutations located in the domain of the ATP pocket (Lynch *et al*, 2004); it is not certain that these mutations impact on the antitumour efficacy of monoclonal antibodies. Taken together, the above-mentioned arguments provided justification for testing the combination of C225 and ZD1839. A recent study by Huang *et al* (2004) examined the antitumour effects resulting from this dual combination more marked tumour regressions were observed with the combination of ZD1839 and C225 in mice bearing a human lung cancer xenograft. Another recent study by Matar *et al* (2004), based on both *in vitro* and *in vivo* data, led to similar conclusions.

In the present study, when combining data from cell survival and those obtained by examining molecular factors, there are strong concording arguments suggesting that the combination of the two drugs triggers less than additive cytotoxic effects. The studies by Huang *et al* (2004) and Matar *et al* (2004) were based on *in vitro* and *in vivo* experiments. It must be underlined that, when examining the different cell lines which were explored in these two latter studies, the supra-additivity of the dual EGFR targeting was not found in all explored cell lines; these cell lines differed markedly between them for the EGFR content. Differences in intrinsic EGFR tumoral expression may modulate the final impact of the dual EGFR targeting and explain the differences between the present conclusions and those reported by the two other groups (Huang *et al*, 2004; Matar *et al*, 2004). In the present study, the infra-additive impact on cell survival was sustained by the changes in cleaved PARP, a faithful molecular indicator of apoptotic process, showing that C225-ZD1839 caused less apoptosis than ZD1839 alone. Further drug-related specific molecular examination indicated that, following drug exposure and cell stimulation by the medium, there was very little activation of the Map kinase pathway (changes in P-p42–44) following cell treatment by either drug, contrasting with the sharp increase in P-p42–44 noted after the combined application to ZD1839 plus C225 (Figure 3c). This observation could plausibly be explained by the fact that C225, markedly, and ZD1839, slightly, downregulate EGFR expression (Figure 4), while their combination has a marked opposite effect with an overexpression of EGFR. Importantly, both analytical methods (Western blot and ligand-binding assay) concurred to highlight the upregulation of EGFR when administering the drug combination. This means that the increase in EGFR involves active and functional receptors (data from Scatchard analysis). The underlying mechanism of this upregulation of the EGFR target produced by the two drugs is not easy to elucidate. Receptor downregulation has been studied most effectively for tyrosine kinase receptor and especially for EGFR (Waterman and Yarden, 2001). Thus, subsequent to its ubiquitination, EGFR is subject to lysosomal degradation (Citri *et al*, 2002). It has been reported that the binding of the natural ligand to EGFR results in a conformational change in the external domain of the receptor (Greenfield *et al*, 1989), which could be crucial to the ligand-induced internalisation of the receptor (Opresko *et al*, 1995). There is thus a ligand-controlled turnover in the expression of EGFR, which could be deregulated in the combined presence of C225 and ZD1839. Recently, the identification of proteins p70 and Clip 4 was reported (Kowanetz *et al*, 2004); these proteins inhibit endocytosis of EGFR and interact with Cbl, a ubiquitin ligase which plays a critical role in EGFR endosomal degradation (Ettenberg *et al*, 2001). It is possible that the combined presence of C225 and ZD1839 may alter the interaction of Cbl with EGFR by a conformational change induced in EGFR.

In conclusion, the present study provides concording pre-clinical data based on cell toxicity and molecular pharmacology. These data suggest that new and tempting treatment strategies on the EGFR target consisting in a double hit with a monoclonal antibody and a tyr kinase inhibitor must be considered with caution.

## Figures and Tables

**Table 1:** Combined cytotoxic effects with C225 and ZD1839

		**CI value for the combination between C225 andZD1839^b^**		
**Cell line**	***R* value for the combination between C225andZD1839^a^**	**50**	**75**	**90**
CAL33	1.7±0.6	1.5±0.05	1.8±0.7	2.6±2.3
CAL39	3.3±1.5	c	1.36±0.2	2.7±2

a *R*, mean±s.d. values from three separate experiments.

b CI mean±s.d. values from three separate experiments for 50, 75, 90% cytotoxic effects.

c Calculation not feasible.

**Table 2:** EGFR quantification (Scatchard analysis)

	**Control**	**ZD1839 (2 × 10^−6^ M)**	**C225 (10^−9^ M)**	**ZD1839+C225**
*N* high-affinity receptors	43.12±0.8	50.19±0.93	39.85±0.74	88.2±1.63
KD	0.31±0.12	0.22±0.11	0.22±0.15	0.48±0.07

Mean±s.d. for EGFR numbers *N* (fmol per well) and dissociation constants *K*_D_ values (nM) determined using Scatchard plot analysis. See details for experimental conditions in the Materials and Methods section.
